# Incidence reporting via online high school concussion surveillance by certified athletic trainers and school nurses, 2015–2018

**DOI:** 10.1186/s40621-019-0228-5

**Published:** 2020-01-13

**Authors:** Lauren Gonzalez, Laura E. Jones, Maryanne Fakeh, Nimit Shah, Joseph A. Panchella, Derek G. Shendell

**Affiliations:** 1 New Jersey Safe Schools Program, Rutgers School of Public Health, 683 Hoes Lane West, 3rd Floor SPH Building, Suite 399 (office 384), Piscataway, NJ 08854-8020 USA; 2 Department of Biostatistics & Epidemiology, Rutgers School of Public Health, 683 Hoes Lane West, Piscataway, NJ 08854 USA; 3 Department of Urban-Global Public Health, Rutgers School of Public Health, One Riverfront Plaza, Suite 1020, Newark, NJ 07102 USA; 4 West Deptford High School Athletics Department, 1600 Old Crown Point Road, West Deptford, NJ 08093 USA; 5 Department of Environmental and Occupational Health, Rutgers School of Public Health, 683 Hoes Lane West, Piscataway, NJ 08854 USA; 6 Environmental and Occupational Health Sciences Institute, Rutgers, The State University of New Jersey, 170 Freylinghuysen Road, Piscataway, NJ 08854 USA; 7 Brain Injury Alliance of NJ-Concussion in Youth Sports Committee, 825 Georges Road, North Brunswick, NJ 08902 USA

**Keywords:** Adolescent health, Athletic trainer, Brain injuries, Concussions, Epidemiology, Incidence, New Jersey, School nurse, Surveillance, Symptoms

## Abstract

**Background:**

There is an increasing concern over adolescent concussions in sports due to risks of long-term negative effects. This study analyzed data over three school years on reported concussion incidence rates by season, high school grade levels and gender, and reported symptoms by school nurses versus athletic trainers, from New Jersey student-athlete concussion data available from an online school-based surveillance system.

**Methods:**

School nurses and athletic trainers reported 300 concussions within five days from when each occurred over three school years, 2015–2018, in team sports and physical education in New Jersey high schools. Analysis was further conducted on symptoms and number of symptoms reported by school nurses versus school athletic trainers for each documented student-athlete concussion. Estimated concussion incidence rates were calculated using state agency verified school enrollment data.

**Findings:**

Concussions most commonly occurred during fall, followed by spring, then winter. Concussion incidence rates ranged from 6.3/1000 (4.99, 7.55) – 9.1/1000 (7.27, 10.98) students over the three school years of the study. Athletic trainers completed 86% of the reports while nurses completed 11% (position or title of 3%, or *n* = 7, were not disclosed); the values were similar when considering only fall pre-season and regular season sports (88, 10, 2%, respectively). On average, across the three school years, athletic trainers reported about 3.5 symptoms per report while nurses reported 2.7 (values in fall seasons only were 3.7 and 3.1, respectively.)

**Conclusions:**

Certified athletic trainers, compared to school nurses, more often completed concussion report forms and reported more symptoms per injured student, perhaps due partly to closer contact and immediate care provided after injury. Additionally, this study had a higher concussion incidence rate during fall sports seasons compared to winter and spring. Future research can further improve our understanding of concussions among adolescent student-athletes to better inform concussion identification, management and recovery protocols.

## Introduction

Concussion, also called mild traumatic brain injury, is of present concern in pediatrics and in sports medicine among adolescents (Halstead and Walter 2010). Concussions can cause acute and chronic adverse effects, as adolescent brains are still developing (Patel and Reddy 2010).

Using 2017 Youth Risk Behavior Survey data, the U.S. Centers for Disease Control and Prevention (CDC) estimated about 15% (2.5 million) of U.S. students had at least one concussion the prior year from either playing a sport or being physically active; 6% reported greater than one concussion (DePadilla et al. 2018). Adolescents and children, compared to adults, sustain the most sports-related concussions (Halstead and Walter 2010). Approximately 300,000 head injuries, 90% of which are concussions, occur among high school (HS) athletes every year (Patel and Reddy 2010). There has been an increase in visits to the emergency department for concussion in the past 10 years: between 2005 and 2009, there were also over two million outpatient medical visits for concussions sustained by U.S. children and adolescents (Lumba-Brown et al. 2018).

Concussion incidence among athletes is underreported for several reasons: signs and symptoms may not be recognized by athletes and coaches, and some athletes may not report head injuries or concussion symptoms to avoid exclusion from play (Patel and Reddy 2010). The ability to rapidly identify concussions in order to initiate care and treatment is crucial. Moreover, there is concern over repeat concussions (Covassin et al. 2013; Taylor et al. 2018; Bruce and Echemendia 2004). Recently, CDC published on the diagnosis and treatment of concussion in children (Lumba-Brown et al. 2018), the first evidence-based (Lumba-Brown et al. 2018; Covassin et al. 2013; Kontos et al. 2012; Merritt and Arnett 2014; Merritt et al. 2015; Lau et al. 2011) guidelines for treatment of concussion for U.S. children. The present study analyzed reported student-athlete concussion data from three consecutive school years (2015–2018), as reported by a certified athletic trainers (ATCs) and school nurses, employed full time, at public secondary or HS throughout New Jersey (NJ) to an online surveillance system (Shendell et al. 2018; Shendell et al. 2019). Numbers of post-concussion symptoms reported within a week of injury were also analyzed to initially compare symptoms reported by school nurses versus school ATCs. We estimated concussion incidence rates by school year and by season for three school years 2015–2018, to expand on previously published descriptive epidemiology (Shendell et al. 2018; Shendell et al. 2019).

## Materials & methods

A pilot study of online concussion reporting in NJ public HS was conducted over the 2015–2016, 2016–2017, and 2017–2018 school years. The study was approved by the Rutgers University Institutional Review Board (#Pro20150001455). Details of the community and school-based process to develop this school-based online surveillance form are available in previous publications (Shendell et al. 2018; Shendell et al. 2019). Consenting, voluntarily participating schools were part of a convenience sample throughout the state of NJ, representing northern, central, and southern regions of the state, for a total of 12 school districts in eight counties. There were at least two school districts per region in each school year of the study.

Concussion injury and incident surveillance data were collected using an online survey form (PsychData LLC). Forms were completed by school nurses or ATCs within five days of a confirmed concussion, which were identified and confirmed by staff or reported by students then verified by staff. Information on demographics, additional injury locations (neck, etc.), cause of injury (person-to-person collision, etc.), personal protective equipment worn (helmet, etc.), symptoms (dizziness, etc.) and activity type were collected. Activity type taking place at time of injury was specifically asked in the 2017–2018 survey, although we were able to infer activity type for all except eleven injuries among HS students in the 2015–2016 and 2016–2017 school years based on personal protective equipment worn and short answer responses by ATCs and school nurses, which provided details and nature of the injury. Details of the other variables, specifically for the 2015–2016 and 2016–2017 school years, are available in previous publications (Shendell et al. 2018; Shendell et al. 2019). Surveillance data were analyzed using Microsoft Excel (Microsoft, Inc.) and SAS (v.9.4, Cary, NC). Descriptive statistics were generated using Excel. Confidence intervals were calculated using SAS. Data were stratified by grade level, gender, school year, and by activity type, e.g., football vs. cheerleading etc.

Additional analysis was conducted to compare numbers of symptoms reported by school ATCs versus school nurses; our prior research documented these groups most often completed forms (Patel and Reddy 2010; Shendell et al. 2019).

Incidence rates and 95% confidence intervals were calculated using concussion data from the pilot study and publically available total school enrollment data from NJ Department of Education (New Jersey Department of Education n.d.). Data on total school enrollment was used as the denominator to calculate incidence rates and confidence intervals for each school year.

## Results

Seven HS with ten campuses participated in 2015–2016, seven HS with seven campuses participated in 2016–2017, and 13 HS with 13 campuses participated in 2017–2018. Overall, 300 concussions were reported over the three years of the pilot study, with 298 concussions among HS students and two concussions among male 8th graders. Concussions sustained by eighth graders were excluded from the incidence analysis but included in the symptom reporting analysis among ATCs and school nurses. There were 113 concussions among HS students and two among 8th graders in 2015–2016, 93 concussions in 2016–2017, and 92 concussions in 2017–2018; these are stratified by school year and grade in Table 1.

**Table 1:** Annual school year reported student concussions during the 2015–2018 school years

Reported Concussions	9th Grade	10th Grade	11th Grade	12th Grade
Total (2015–2018)	84	89	74	51
2015–2016 school year only	39	34	27	13
2016–2017 school year only	30	27	21	15
2017–2018 school year only	15	28	26	23
*Annual average # concussion injuries reported, 2015–2018*	*28*	*30*	*25*	*17*

The average age of HS students at the time of concussion was approximately 15–16 years (15.6 for male students and 15.5 for female students). HS students were predominantly white (74%) and non-Hispanic (82%); 176 (59%) were male and 122 (41%) were female (Table 2).

**Table 2:** Demographics of student-athletes with concussions in the 2015–2018 school years ^a^

	2015–2018 school years combined
Gender, n (%)	
Male	178^b^ (59.7)
Female	122 (40.9)
Race, n (%)	
White	221 (74.2)
Black	41 (13.8)
Asian	14 (4.7)
Other^c^	23 (7.7)
Ethnicity, n (%)	
Hispanic/Latino	50 (18.0)
Non-Hispanic/Latino	228 (82.0)

^a^ Note: Some categories smaller due to non-response; some percentages do not add up to 100 due to rounding

^b^ Two of the male concussions were sustained by students in 8th grade

^c^ Other races include American Indian/Alaska Native, Native Hawaiian/Pacific Islander, and any races defined as “other” by the respondent

**Fig. 1:**
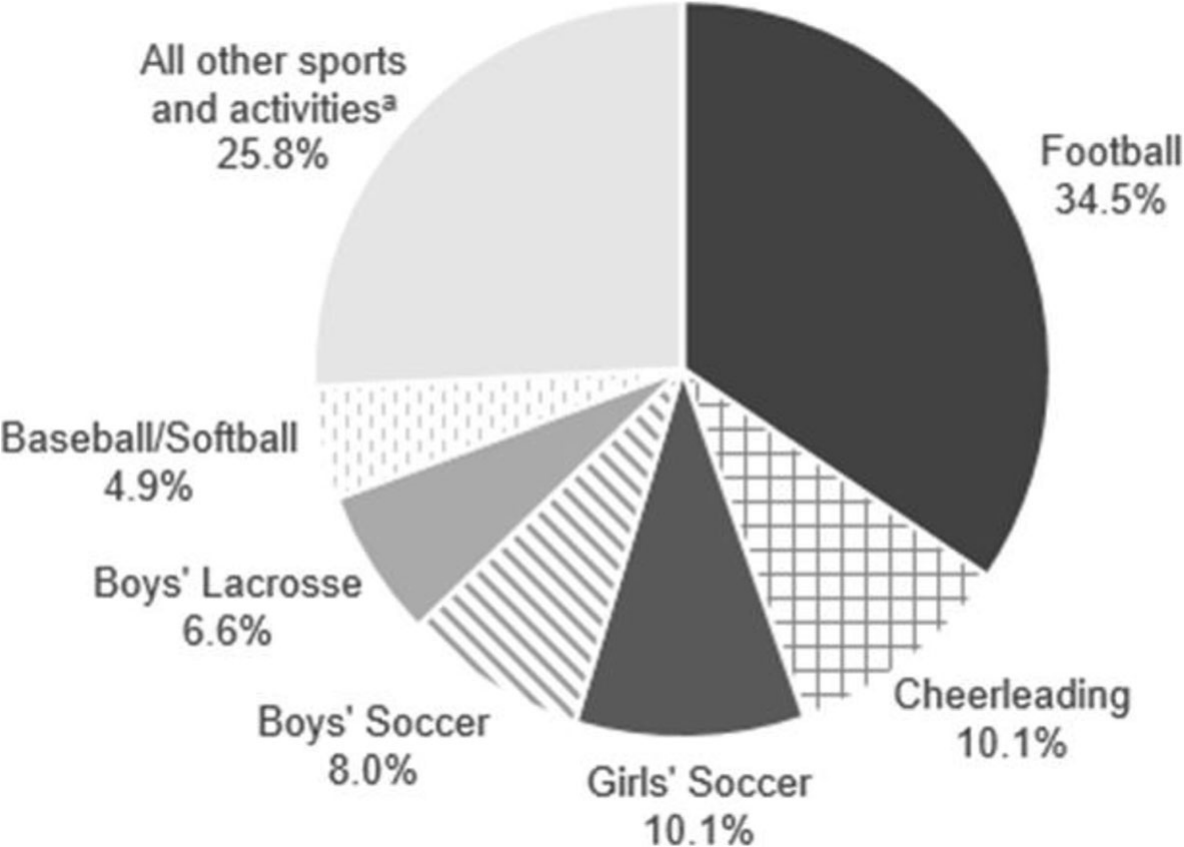
Reported concussions by activity type (of total of 298 for 9th–12th graders) during 2015–2018 school years. ^a^Note “all other sports and activities” included wrestling, field hockey, girls’ lacrosse, boys’ basketball, girls’ basketball, physical education, volleyball, golf, JROTC Basic Training, track and field, and instances where activity type data were missing

Among HS students, the sport or activity in question was reported or able to be determined for 287 of 298 concussions (Fig. 1). There were 99 concussions (35%) which occurred during football play or practice; 29 concussions (10%) were sustained during girls’ soccer; and 29 (10%) were sustained during cheerleading (although co-ed, concussions were only reported among girls). Additionally, 23 concussions (8%) occurred during boys’ soccer, and 19 (7%) occurred during boys’ lacrosse. The remaining concussions occurred during other sports, physical education, other activities, or when activity type was not reported. At least one concussion was reported for football, cheerleading, field hockey, boys’ soccer, girls’ soccer, volleyball, wrestling, boys’ basketball, girls’ basketball, baseball/softball, boys’ lacrosse, girls’ lacrosse, track and field, physical education including Junior Reserve Officer Training Corps (JROTC) Basic Training, golf, and other activities (Shendell et al. 2018; Shendell et al. 2019).

Overall, among HS male student-athletes, football contributed to 93 (55%) of the 170 concussions, for which activity type data were available. Additionally, boys’ soccer contributed to 23 concussions (14%) and boys’ lacrosse contributed to 18 (11%). When analyzed without football, the sport breakdown for male athletes (*n* = 77) included 23 concussions from soccer (30%), 18 from lacrosse (23%), and 11 from wrestling (14%). Among female student athletes, 29 concussions (25%) occurred during cheerleading, 29 (25%) occurred during girls’ soccer, and 10 (9%) occurred during softball.

Over the three years of this pilot study, ATCs and nurses completed 300 concussion reports; 86% of reports were completed by ATCs (*n* = 259), school nurses completed 11% (*n* = 34), and 2% did not complete reporting form job title and symptom fields (n = 7). In total, 961 symptoms were reported among the 300 reported concussions, for an average of 3.1 symptoms reported per concussion. The ATCs reported, on average, more symptoms associated with reported student-athlete concussions than the school nurses, 3.5 versus 2.7, respectively; the values for the combined summer pre-season and fall regular season were slightly higher at 3.7 for ATC and 3.1 for school nurses, respectively (Table 3).

**Table 3:** Number of symptoms reported per concussion injury by school nurses versus athletic trainers for 300 student-athlete concussions documented in the 2015–2018 school years

	By Nurses		By Athletic Trainers	
Number of Symptoms Reported	n	%	n	%
0	4	12%	19	7%
1	5	15%	33	13%
2	7	21%	54	21%
3	7	21%	60	23%
4	4	12%	32	12%
5	2	6%	22	8%
6	4	12%	13	5%
7	1	3%	11	4%
8+	0	0%	15	6%

Nevertheless, both groups (ATCs and school nurses) had similar distributions of numbers of symptoms reported, with a range 1–7 symptoms reported for about 88% of report forms and about 22% of report forms noting either two symptoms or three symptoms per student-athlete, respectively. Dizziness, headaches, and sensitivity to indoor and/or outdoor lighting (artificial or via sunlight) were consistently documented on 20% or more of reported concussions. Although feeling disoriented, blurry vision, and balance issues were reported frequently among ATCs, (range 20–27% of reported concussions), school nurses did not report these symptoms as frequently (6–15% range); it should be noted, however, among school nurses, sensitivity to noise and sensitivity to indoor artificial lighting ranked co-third (21% each of reported concussions). Both groups reported fatigue and nausea in a similar proportion (12–15%). (See Additional file 1: Table S1).

Concussion incidence rates ranged from 6.3/1000 to 9.1/1000 enrolled HS students over the three study school years (Table 4, presented with 95% confidence intervals). Concussions most commonly occurred during fall, followed by spring, then winter. Given the 95% confidence intervals overlapped, overall rates varied slightly but were not statistically significantly different across the three study years.

**Table 4:** Concussion incidence rates by season and by year

Season/School year	Number of concussions	Concussions/1000 enrolled HS students 95% confidence intervals
Fall 2015	86	6.5 (5.12,7.87)
Winter 2015–2016	7	0.5 (0.14, 0.92)
Spring 2016	22	1.7 (0.97, 2.36)
Total in 2015–2016 school year	115	8.7 (7.10, 10.27)
Fall 2016	56	5.5 (4.06, 6.93)
Winter 2016–2017	15	1.5 (0.73, 2.22)
Spring 2017	22	2.2 (1.26, 3.06)
Total in 2016–2017 school year	93	9.1 (7.27, 10.98)
Fall 2017	76	5.2 (4.01, 6.34)
Winter 2017–2018	9	0.6 (0.21, 1.01)
Spring 2018	7	0.5 (0.12, 0.83)
Total in 2017–2018 school year	92	6.3 (4.99, 7.55)

## Discussion

In 2016, CDC proposed a national concussion surveillance system (Centers for Disease Control and Prevention (CDC) n.d.). This study is one of multiple attempts to address gaps in youth concussion and symptom surveillance in the U.S. (Kerr et al. 2017; O'Connor et al. 2017) Also in 2016, a statewide registry based at the University of Texas – Southwestern was established to track concussions among student-athletes in Texas; it was the largest statewide effort to date to track concussions among student-athletes (UT Southwestern Medical Center 2018). Both NJ (Shendell et al. 2018; Shendell et al. 2019) and Texas (UT Southwestern Medical Center 2018) could serve as potential models for similar systems in other states as well as for the CDC’s proposed national system.

Overall, there were more concussions during the fall sports seasons, likely because of the high proportion of male student athletes participating in freshman, junior varsity, and varsity football (Shendell et al. 2018; Shendell et al. 2019). Participating ATCs noted there were generally more students participating in fall sports and how, in general, there was no upper limit on the number of students able to join the sports teams during this season. Thus, there were likely more student-athletes during the fall sports season, and consequently more student-athletes at risk for concussion.

The present study’s data suggested the most reported HS student-athlete concussions occurred during the fall, and reported in football, which was associated with 35% of total concussions for which activity type information was available. This information reinforces other work in the literature which demonstrated both HS and college football players become concussed at higher rates than players of other sports such as softball (Gessel et al. 2007). One of the important findings of the present study was how the sport of cheerleading, which is increasing in popularity and numbers of participants across the U.S. due to its athletic and energetic gymnastics, acrobatic, and dance-related components, ranked “top 3” in each study school year and in each fall and winter sports season (Cheer Safe 2019; UCA 2019; Varsity 2019). The present study thus informs ATCs and coaches in the state of NJ and across the U.S. about cheerleading concussions, and reinforces the message to be particularly vigilant during the fall sports season. In 2011, the State of NJ mandated public school districts, charter schools, and private schools adopt an interscholastic head injury training program for coaches, ATCs, school nurses, and other appropriate personnel (N.J.S.A. 18A:40–41.4 n.d.). The content of the program is at the discretion of the individual school districts provided it meets minimum requirements, including identifying the signs and symptoms of concussions and a graduated return-to-play protocol for concussed student-athletes. Further information on specific sports where student-athletes could be at higher risk of concussion, based on the present study, may enhance these training programs.

There were more reported concussions among 9th and 10th graders, i.e., underclassmen, as compared to 11th and 12th graders, i.e., upperclassmen. This finding may be due to how, in general, freshman and junior varsity teams often allow more and younger students to participate, and usually do not cut players, unlike typical varsity teams.

In New Jersey, a survey reported about 95% of public secondary schools had certified athletic training services, of which 91% served the school full time (Pryor et al. 2015). For comparison, 70% of U.S. public secondary schools had athletic training services, although only 37% of them served the schools full-time (Pryor et al. 2015; Adams et al. 2019), and among public and private U.S. secondary schools combined about 58% had athletic training services, with 28% full-time (in NJ, 68 and 36%, respectively) (Pike et al. 2017; Pike et al. 2016). The present study, which had a greater involvement by certified ATCs than school nurses, documented a small difference in reporting symptoms by school nurses versus school ATCs. On average ATCs reported more concussions compared to school nurses. Additionally, while dizziness and headaches, the top two reported symptoms, were the same, there was variation in the other symptoms reported, which may be attributable to many factors, including the symptoms which the nurse or athletic trainer were able to see at time of injury or time of reporting. It must be noted this study did not follow-up with these students over time.

Concussion incidence rates by gender were not calculated in this study due to the lack of gender-specific denominator data; however, current literature has suggested female student-athletes tended to have higher concussion rates than male student-athletes when playing similar sports (Halstead and Walter 2010; Kerr et al. 2017; O'Connor et al. 2017), although male student-athletes sustained more concussions overall (Halstead and Walter 2010; Resch et al. 2017). Therefore, analysis of concussion rates by gender, are worthy of further study, with more symptoms assessed.

This study had limitations. First, it was a pilot project with a limited sample size, i.e., a sample of participating, consenting school districts and HS. Second, only total school enrollment data were available, not team rosters, which allowed only for calculations of season-level and yearly estimated incidence rates. Third, the online reporting form was filled out by a school nurse or athletic trainer, meaning evaluations of reported concussions were, theoretically, more consistent based on the NJ-mandated training on sports-related concussions, plus personal professional experience. However, data were only collected by participants at one time point for each subject. Fourth, only the 2017–2018 school year questionnaire specifically asked what activity students were engaging in while they sustained a concussion. Information on activity type from 2015 to 2016 and 2016–2017 was obtained from the free-response sections where the respondent described what occurred when the student sustained a concussion. Finally, there was no way of determining if a concussion injury occurred at a participating HS but went unreported, and assessing any difference in concussion severity by who the injury was reported to/by (for entry into this study’s online surveillance tool) was beyond the scope of this study..

It should be noted the first two years (2015–2017 school years) contained several free-response sections, which led to inconsistent data. For example, activity type at the time of the concussion injury was determined from the free-response narrative sections describing the concussion injury incident during the 2015–2016 and 2016–2017 school years. The reporting form for the final year (2017–2018 school year) included more multiple choice-style questions, which led to more consistent reporting and may have reduced the time necessary to complete the form.

This study had known strengths. This online surveillance form proved to be easy-to-use, low- or no-cost, and able to be completed rapidly (Shendell et al. 2018; Shendell et al. 2019). The NJ Department of Education’s recent online public release of school enrollment data allowed for the calculation of estimated concussion incidence rates for the first time using this dataset. While there is always concern for underreporting of concussion by student-athletes themselves, concussion injury events in this study were identified by staff or reported by students. It is therefore unlikely the participating school nurse or certified athletic trainer would have deliberately completed a surveillance form incorrectly. When they logged into the online form, they were acknowledging the specific concussion injury assessed and documenting known circumstances surrounding the incident in question.

While many states (Centers for Disease Control and Prevention (CDC) n.d.) and the CDC (Centers for Disease Control and Prevention (CDC) 2015) have recently made efforts to monitor and address youth concussions, especially among student-athletes, more work remains to be done. Reporting is inconsistent, often university-based studies with variable sample sizes and which may not be representative of most student-athletes, e.g., only among male student-athletes (Long et al. 2018), or only among soccer players (Nevins et al. 2018).

## Conclusion

This analysis suggested student-athletes sustained concussion injuries throughout the school year and while participating in various sports and activities sponsored by public secondary schools. An online reporting form was able to yield useful epidemiological data on concussion injury occurrence. In this study, football players comprised many of the total reported incidents, overall and particularly in fall seasons, but student-athletes participating in other sports such as soccer (fall seasons) and cheerleading (fall and winter seasons) also played a large part in these reported concussions. In future studies, the plan is to accurately determine sizes of the participating and most at-risk school teams to get sports-specific estimated concussion rates during practice and competition. Guidelines for concussion management in adults (ages 16 and older) have been available since 2008 from the American College of Emergency Physicians (ACEP 2008); guidelines for youth concussion were released by CDC in 2018 (Lumba-Brown et al. 2018). Increased state-level and national surveillance of youth concussion to complement clinical guidelines is a public health priority.

The need for full- time and accessible school appointed health-care professionals to the student body, which includes the student athletes, is crucial. ATCs are commonly available after school hours when play typically takes place as opposed to school nurses, who may only be able to see the student during school hours.

## Supplementary information

**Additional file 1: Table S1.** School nurse versus athletic trainer symptom reporting for 300 student-athlete concussions documented in the 2015–2018 school years

## Data Availability

The surveillance datasets generated during the study are confidential and protected information in accordance to IRB protocol, datasets analyzed during the current study to determine incidence rates are available via the NJ Department of Education, https://www.state.nj.us/education/data/enr/

## Abbreviations

ATCs: Certified Athletic Trainers
CDC: U.S. Centers for Disease Control and Prevention
HS: High School
NJ: New Jersey
