# Supplementary material for: Incidence reporting via online high school concussion surveillance by certified athletic trainers and school nurses, 2015–2018
Source: Inj Epidemiol. 2020 Jan 13;7:1. doi: 10.1186/s40621-019-0228-5 (PMC6956499; doi:10.1186/s40621-019-0228-5)
Supplement: Supplementary file 1 — Additional file 1: Table S1. School nurse versus athletic trainer symptom reporting for 300 student-athlete concussions documented in the 2015–2018 school years [file 40621_2019_228_MOESM1_ESM.docx]

**Supplementary Material**

Additional file 1: Table S1. School nurse versus athletic trainer symptom reporting for 300 student-athlete concussions documented in the 2015-2018 school years

|  | Reported by Nurses | | Reported by Athletic Trainers | |
| --- | --- | --- | --- | --- |
| Symptoms | n times | % | n times | % |
| Balance Issues | 4 | 12% | 69 | 27% |
| Blurry Vision | 2 | 6% | 51 | 20% |
| Dizziness | 17 | 50% | 143 | 55% |
| Fatigue | 4 | 12% | 33 | 13% |
| Feeling Disoriented | 4 | 12% | 66 | 25% |
| Headache ` | 25 | 74% | 222 | 86% |
| Nausea | 5 | 15% | 34 | 13% |
| Neck pain | 5 | 15% | 53 | 20% |
| Numbness and/or tingling in hands or feet | 1 | 3% | 3 | 1% |
| Ringing in one or both ears | 0 | 0% | 19 | 7% |
| Seeing Stars | 1 | 3% | 22 | 8% |
| Sensitivity to indoor artificial lighting | 7 | 21% | 40 | 15% |
| Sensitivity to noise | 7 | 21% | 24 | 9% |
| Sensitivity to outdoor artificial (stadium or field) lighting | 3 | 9% | 12 | 5% |
| Sensitivity to outdoor sunlight | 5 | 15% | 41 | 16% |
| Slowed Speech | 0 | 0% | 7 | 3% |
| Vomiting | 0 | 0% | 4 | 2% |
| Other | 6 | 18% | 22 | 8% |
| None | 0 | 0% | 1 | 0% |
| Unknown | 4 | 12% | 16 | 6% |
